# Active Commuting to and from School, Cognitive Performance, and Academic Achievement in Children and Adolescents: A Systematic Review and Meta-Analysis of Observational Studies

**DOI:** 10.3390/ijerph16101839

**Published:** 2019-05-23

**Authors:** Abel Ruiz-Hermosa, Celia Álvarez-Bueno, Iván Cavero-Redondo, Vicente Martínez-Vizcaíno, Andrés Redondo-Tébar, Mairena Sánchez-López

**Affiliations:** 1 Social and Health Care Research Center, Universidad de Castilla-La Mancha, c/ Santa Teresa Jornet, s/n, 16071 Cuenca, Spain; Abel.RuizHermosa@uclm.es (A.R.-H.); Ivan.Cavero@uclm.es (I.C.-R.); Vicente.Martinez@uclm.es (V.M.-V.); Andres.Redondo@uclm.es (A.R.-T.); Mairena.Sanchez@uclm.es (M.S.-L.); 2 School of Education, Universidad de Castilla-La Mancha, Ronda de Calatrava, 3, 13071 Ciudad Real, Spain; 3 Facultad de Ciencias de la Salud, Universidad Autónoma de Chile, Av. Pedro de Valdivia 425 (Sede Santiago), Providencia 7500000, Chile

**Keywords:** active transportation, active travel, walking, cycling, physical activity, exercise, cognition, academic performance, youth, school performance

## Abstract

Background: Physical activity has a beneficial effect on the brain’s development process and cognitive function. However, no review to date has evaluated the effects of active commuting to and from school (ACS) on cognitive performance and academic achievement. The aim of this systematic review and meta-analysis was to evaluate the link between ACS and cognitive performance and academic achievement in children and adolescents. Methods: We systematically searched MEDLINE, EMBASE, Web of Science and PsycINFO databases for all observational studies published until May 2019 that examined the association between ACS and cognitive performance or academic achievement. Studies were classified into two groups according to their measured outcomes: cognitive performance (nonexecutive cognitive functions, core executive functions, and metacognition) and academic achievement (marks of different areas). A pooled effect size (ES) was estimated using the DerSimonian and Laird random-effects method for cognitive performance and each area of academic achievement. Results: Twelve studies that evaluated the relationship between ACS and cognitive performance or academic achievement were included in the systematic review: four studies analyzed both cognitive performance and academic achievement, one study provided data regarding cognitive performance and seven provided data on academic achievement. Finally, nine of 12 studies provided enough data for inclusion in the meta-analysis. Our findings suggest that ACS was not significantly associated with cognitive performance (ES = −0.02; 95% CI: −0.06 to 0.03) or academic achievement (ES = −0.33; 95% CI: −0.83 to 0.17 for mathematics-related skills; ES = −0.37; 95% CI: −0.88 to 0.15 for language-related skills). Conclusions: There was insufficient evidence regarding the relationship between ACS and cognitive performance and academic achievement. Future studies should include potential confounders in their analyses and consider the use of standardized self-reports or objective measures of ACS.

## 1. Introduction

It is widely documented that physical activity (PA) has a positive impact on diminishing the risk of some cardiometabolic diseases such as obesity, diabetes, or coronary disease, and mortality [1,2]. However, regular participation in PA is a lifestyle factor that can provide numerous benefits beyond physical health. In the last decade, a growing body of literature has suggested that PA has a beneficial effect on the brain’s development processes and cognitive function in children and adolescents, thus leading to better learning and academic achievement [3,4,5,6,7]. In addition, some studies have suggested that PA could reduce the risk of mental health disorders (such as stress, depression and low self-esteem) [4,8] and improve various life skills, including decision-marking, problem solving or creativity [9,10]. Different pathways or mechanisms by which regular PA appears to exert this beneficial effects on the brain have been proposed, including increased blood flow and oxygen to the brain, improved neurogenesis, and synaptic plasticity, as well as increased levels of norepinephrine and endorphins [11,12,13]. Moreover, previous studies have suggested that PA increased certain growth factors (such as brain-derived neurotrophic factor or insulin-like growth factor-1) that improve the function and structure of the brain [11,14]. Thus, through these mechanisms, regular PA may improve the schoolchildren’s behavior in the classroom, leading to better concentration on and attention to the academic content [11]. Nevertheless, despite these benefits, a growing number of studies have reported that most children and adolescents do not meet current PA recommendations [15,16,17,18,19].

It is known that the exercise at early age (during brain development) produces more benefits than at later ages [20]. In addition, this is a key period of life in which individuals develop and consolidate healthy habits that tend to persist over time, since the adolescent brain is sensitive to be influenced by environment and modifiable lifestyle behaviors such as PA [21,22]. However, unfortunately, the adolescence is a period of life where PA levels drop drastically [23].

One way to increase levels of daily PA is to integrate active commuting to and from school (ACS) by walking, cycling or skateboarding into the daily routine of children and adolescents [24]. Recent studies have shown that ACS represents approximately 23% of the time spent in PA per week [25] and can result in up to 45 additional minutes of daily moderate-vigorous PA [26]. Furthermore, previous systematic reviews have indicated that usual ACS is associated with healthier body fat and higher values of cardiorespiratory fitness, especially in children and adolescents who cycle to and from school [26,27]. However, although several authors have pointed out the need to examine which strategies and types of PA are most effective in improving cognition [4,7,28], no review to date has evaluated the association of ACS on cognitive performance and academic achievement. Given that previous studies have suggested that regular PA has positive effects on cognitive performance and academic achievement [5,6], habitual ACS (as a way to increase the amount of daily PA) may be positively related with these improvements in children and adolescents, although consistent evidence about this is lacking. In addition, given that several inconsistencies regarding the assessment of the relationship between PA and both cognitive performance and academic achievement outcomes [3,29] have been reported, it seems relevant to analyze the relationship to ACS while distinguishing between cognitive performance and academic achievement.

Thus, the aim of this systematic review and meta-analysis was to examine the association between ACS and both cognitive performance and academic achievement in children and adolescents.

## 2. Materials and Methods

### 2.1. Definitions

For the purposes of maintaining clarity and consistency in this review, definitions for the following terms are provided:

- Active commuting to and from school. Is defined as the use of active means of transportation as walking, cycling, skateboarding, or other nonmotorized means that implies energy expenditure for commuting to and from school.
- Cognitive performance. In this review, cognitive performance is used to describe the cognitive functions of schoolchildren through performance of standardized and validated tests that including different components of cognition [30]. Following the classification adopted in a recent systematic review and meta-analysis [5], we established three groups of cognitive functions: (1) nonexecutive cognitive functions (cognitive domains related minimally with executive function, such as decision making or processing speed) [5]; (2) core executive functions (mental processes that generally include three core executive functions: inhibition, working memory and cognitive flexibility) [31]; and (3) metacognition (the individual´s capacity to understand cognitive processes and use knowledge to regulate behaviors [reflects the use of higher-level executive functions such as planning, reasoning and problem solving]) [29].
- Academic achievement. This term is used to describe the performance of children through the use of standardized tests at school or the educational environment (such as the scores on specific subjects or classroom test scores, the grade point average or other formal assessments) [30].

### 2.2. Registration and Protocol

This systematic review and meta-analysis was registered on 5/12/2017 with the International Prospective Register of Systematic Reviews (PROSPERO) database (Ref. CRD42017079726) and was performed according to the Meta-analysis of Observational Studies in Epidemiology (MOOSE) statement (see supplementary material Table S1 for MOOSE checklist for meta-analysis of observational studies) [32] and the Cochrane Collaboration Handbook [33].

### 2.3. Search Strategy

The literature search was conducted by two reviewers using MEDLINE (via PubMed), EMBASE, Web of Science and PsycINFO databases for all observational studies published until May 2019 that examined the association between ACS and cognitive performance or academic achievement. The search strategy was applied to all the titles, abstracts and keywords of the studies combining the following relevant terms: (1) “commuting”, “active commuting”, “active commuting to school”, “active commuting from school”, “active transportation to school”, “active transportation from school”, “walk*”, “walking to school”, “walking from school”, “cycling”, “cycling to school”, “cycling from school”, “bicycling”, “bicycling to school”, “bicycling from school”, “skateboarding”, “skateboarding to school”, “skateboarding from school” and “lifestyle habit*”; (2) “cognition”, “executive”, “executive function”, “academic”, “academic skill*”, “academic achievement”, “academic performance”, “academic behavior*”, “academic grade*”, “cognitive performance”, “cognitive control”, “cognitive flexibility”, “intelligence”, “memory”, “attention”, “mathematic performance”, “inhibitory control”, “working memory”, “decision making” and “metacognition”; (3) “children”, “childhood”, “preschooler”, “schoolchildren”, “preadolescent”, “adolescent*” and “adolescence” (see supplementary material Table S2 for the MEDLINE database search strategy). To complement the literature search, the reference lists of the articles included in this review were reviewed for possible inclusion.

### 2.4. Study Selection Criteria

Studies were considered eligible if they (1) examined healthy children or adolescents from 4 to 18 years of age, (2) included objective measures or self-report questions to assess ACS, (3) included data differentiated between active and passive commuters in their analysis, (4) included data about the association between commuting to and from school with at least a measure of cognitive performance or academic achievement as a dependent variable, (5) used a cross-sectional design or showed baseline measurements of cohort studies and randomized control trials, and (6) were published or accepted for publication in a peer-reviewed journal. Studies were excluded when the age of the population was outside the specified range or when the target population was specifically children or adolescents with mental disorders that could limit generalizability. Finally, studies were likewise excluded when they were not published in English or Spanish.

### 2.5. Search Data Extraction

Two researchers independently screened all titles and abstracts of the retrieved studies, removing duplicates and excluding those that did not meet the selection criteria. The same two authors independently collected the following data from each selected article: (1) year of publication, (2) country of the study, (3) study design, (4) population characteristics (sample size and age), (5) assessment of ACS (indicator and categories of ACS), and (6) tools and/or scales used for the schoolchildren’s cognitive performance and academic achievement assessment and domains evaluated (Table 1). In addition, the main outcomes and covariates included in each study were also extracted (Table 2 for cognitive performance outcomes, and Table 3 for academic achievement outcomes). The authors of the included studies were contacted when a lack of data was detected. Disagreements among the researchers in the collection of information were resolved by consensus or by involving a third researcher.

Studies were classified into two groups according to their measured outcomes: cognitive performance (distinguishing between nonexecutive cognitive functions, core executive functions, and metacognition) and academic achievement (distinguishing between the marks of different areas and grade point average (self-reported or measured by the teacher).

### 2.6. Risk of Bias

After concealing information about the studies (authors, affiliations, date and sources of manuscript) that met the inclusion criteria, two reviewers independently assessed the methodological risk of bias of the studies (see supplementary material Table S3). The criteria for assessing risk of bias were created based on the STrengthening the Reporting of OBservational studies in Epidemiology (STROBE) criteria [34] and the Effective Public Health Practice Project (EPHPP) [35]. A risk of bias score was calculated based on the following five criteria, employed by Smith and Madden [36] and Rodriguez-Ayllon et al. [37]: (1) adequate description of the study sample (number of participants, mean age and sex); (2) adequate assessment/reporting of ACS (ACS measurement was clearly defined and validated, and the studies included at least three of the following data: duration/distance, intensity, frequency or analysis separating walking, cycling or other means of commuting to and from school); (3) adequate assessment of the cognitive performance and academic achievement outcomes (validity/reliability of the outcome measure reported and/or measurement procedure adequately described); (4) adequate adjustment of confounders (the studies considered at least three of the following confounding variables: sex, age, familial socioeconomic status, distance or total PA); and (5) description of both the numbers and reasons for withdrawals and dropouts (participation rate at baseline at least 70%). Based on previous methodology, the scores were summed to provide a total score out of 5, using the following categories: 0–2 “high risk”, 3 “medium risk”, and 4–5 “low risk”. Disagreements were resolved by consensus or by involving a third researcher.

### 2.7. Statistical Analysis

Only those studies that included data about differences between active and passive travelers on cognitive performance or academic achievement were included in the meta-analysis. Effect sizes (ES) were estimated to depict the relationship between the mode of commuting to and from school for each observation using Cohen´s d index [38]. A pooled ES was estimated using the DerSimonian and Laird random-effects method [39] on cognitive performance and each area of academic achievement in which positive ES values indicated higher outcomes scores in favor of the ACS groups. Heterogeneity across studies was assessed using the I^2^ statistic and was categorized as not important (0% to 40%), moderate (30% to 60%), substantial (50% to 90%) and considerable (75% to 100%) [33]. In addition, the corresponding p values were also considered.

**Table 1:** Characteristics of the included studies.

Study ^a^		Population Characteristics	Outcome		
Reference	Country	Sample Size and Age	ACS ^b^	Cognitive Performance	Academic Achievement
Ruiz-Hermosa et al. 2018 [40]	Spain	1159 (599 boys), 5.3 ± 0.6 (years)	✓ Indicator: usual walking from home to school ✓ Categories: - Non-ACS - ACS	Battery of General and Differential Aptitudes (BADyG): ✓ Nonexecutive functions: - General nonverbal intelligence - Spatial factor ✓ Metacognition: - Logical reasoning ✓ Overall cognitive performance	Battery of General and Differential Aptitudes (BADyG): ✓ Mathematics-related skills ✓ Language-related skills
García-Hermoso et al. 2017 [25]	Chile	389 (196 boys), 12.0 ± 0.6 (years)	✓ Indicator: usual walking to and from school (at least one of the trips must be walking to be considered to be ACS) ✓ Categories: - Non-ACS - ACS ≤ 30 min - ACS 30–60 min - ACS > 60 min - Non-ACS		Grade scores: ✓ Mathematics-related skills ✓ Language-related skills
Mora-González et al. 2017 [41]	Spain	489 (240 boys), 10 ± 1.2 (years)	✓ Indicator: usual walking and cycling to and from school (at least one of the trips must be walking or cycling to be considered to be ACS) ✓ Categories: - Non-ACS - ACS		Grade scores: ✓ Mathematics-related skills ✓ Language-related skills ✓ English language ✓ Natural sciences ✓ Social sciences ✓ Overall academic achievement
		1649 (820 boys), 14.2 ± 1.3 (years)			
Ruiz-Ariza et al. 2017 [42]	Spain	1006 (428 boys), 14.4 ± 1.7 (years)	✓ Indicator: at least 5 trips walking of more than 15 min weekly ✓ Categories: - Non-ACS - ACS (mean of 18.30 min/day)		Grade scores: ✓ Mathematics-related skills ✓ Language-related skills ✓ Physical education ✓ Overall academic achievement
Domazet et al. 2016 [43]	Denmark	568 (269 boys), 13 ± 0.6 (years)	✓ Indicator: usual walking and cycling to and from school ✓ Categories: - Non-ACS - Walking - Cycling	Eriksen flanker task: ✓ Core executive functions: - Inhibitory control (reaction time and accuracy)	Danish Ministry of Education Test: ✓ Mathematics-related skills
López-Vicente et al. 2016 [44]	Spain	2897 (599 boys), 8.6 ± 0.9 (years)	✓ Indicator: Usual walking and cycling from home to school ✓ Categories: - Non-ACS - ACS 1–25 min - ACS 1–25 min - ACS > 25–50 min - ACS >50 min	N-back task: ✓ Core executive functions: - Working memory Attentional network task: ✓ Core executive functions: - Attention	
Martins et al. 2016 [45]	Portugal	391 (189 boys), 16.0 ± 1.5 (years)	✓ Indicator: usual walking and cycling to and from school ✓ Categories: - Non-ACS - ACS one-trip - ACS two-trips (mean of 11.17 min/day)		Self-reported question of academic achievement: ✓ Language-related skills ✓ Mathematics-related skills ✓ Physical education
Van Dijk et al. 2014 [46]	The Netherlands	270 (143 boys), 13.4 ± 1.3 (years)	✓ Indicator: mean moving time (minutes) on weekdays between 7:00 a.m. and 8:40 a.m. x 2 (round-trip) (mean of 46.16 min/day)	D2 test of attention: ✓ Core executive functions: - Response inhibition/selective attention Symbol Digit Modalities test: ✓ Core executive functions: - Information-processing speed	Grade scores: ✓ Overall academic achievement (language-related skills, mathematics-related skills, and English language) ✓ Mathematics-related skills
Stea and Torstveit 2014 [47]	Norway	2432 (1187 boys), 16 ± 0.4 (years)	✓ Indicator: usual walking and cycling to and from school ✓ Categories: - Non-ACS - ACS		Grade scores: ✓ Overall academic achievement (language-related skills, English language, and mathematics-related skills)
Haapala et al. 2014 [48]	Finland	186 (107 boys), 7.7 ± 0.4 (years)	✓ Definition: minutes/day of walking and cycling to and from school ✓ Categories: - Non-ACS - ACS (mean of 18.7 min/day)		Ala-asteen lukutesti (ALLU test battery): ✓ Reading fluency ✓ Reading comprehension Basic Arithmetic test: ✓ Mathematics-related skills
Stock et al. 2012 [49]	Denmark	10,380 (5086 boys), 14.1 ± 0.4 (years)	✓ Indicator: Everyday walking, cycling or skating to and from school ✓ Categories: - Non-ACS - ACS (mean of 14 min/day)		Self-reported question of academic achievement: ✓ Overall academic achievement (very good, good, average, and not good)
Martínez-Gómez et al. 2011 [50]	Spain	1700 (808 boys), 15.4 ± 1.3 (years)	✓ Indicator: usual walking and cycling from home to school ✓ Categories: - Non-ACS - ACS - ACS ≤ 15 min - ACS > 15 min	Short Test of Educational Ability (SRA): ✓ Metacognition: - Reasoning ability ✓ Overall cognitive performance	Short test of Educational Ability (SRA): ✓ Language-related skills ✓ Mathematics-related skills

^a^ All the studies were cross-sectional design, except for López-Vicente et al. [44] and Haapala et al. [48] that were follow-up studies. ^b^ All the studies used self-report questions or diaries to assess ACS, except for Van Dijk et al. [51] that used accelerometers. Abbreviations: ACS, active commuting to and from school.

**Table 2:** Main outcomes of included studies that analyzed the association between active commuting to and from school (ACS) and cognitive performance.

Reference	Results	Covariates
Ruiz-Hermosa et al. 2018 [40]	No differences were found between walking to school and passive commuters with Nonverbal Intelligence and General Intelligence outcomes in children aged 4 to <6 years old. Walking to school was not associated with Logical Reasoning, Spatial Factor and General Intelligence outcomes in children aged ≥6 to 7 years old.	Age, BMI, CRF, and SES.
Domazet et al. 2016 [43]	Walking and cycling to and from school was not associated with Inhibitory Control.	Age, sex, SES, breakfast consumption, and supporting teaching outside the classroom during school hours.
López-Vicente et al. 2016 [44]	No differences were found between active commuting to school and passive commuters in Working Memory and Attention outcomes.	Sex, maternal education, SES, residential neighborhood, and air pollution.
Van Dijk et al. 2014 [46]	ACS was positively associated with executive functioning (Response Inhibition/Selective Attention) in girls (β = 0.17, *p* = 0.037), but not in boys. No differences between ACS and passive commuters with Information-processing Speed outcomes were shown.	Sex, academic year, SES, BMI, depressive symptoms, ethnicity, school level, and PA per week by accelerometer.
Martínez-Gómez et al. 2011 [50]	Girls in the active commuting to school group had significantly higher scores than girls in the non-active commuting to school group in Overall Cognitive Performance (53.20 ± 14.01 vs. 49.61 ± 12.24; *p* < 0.001). In addition, girls in the active commuting to school > 15-minute group had better scores in Reasoning Ability and Overall Cognitive Performance (*p* < 0.05) than girls in the active commuting to school ≤ 15-minute group. No significant differences were found in boys.	Age, school, BMI, and extracurricular PA.

Abbreviations: BMI, body mass index; CRF, cardiorespiratory fitness; PA, physical activity; SES, socioeconomic status.

**Table 3:** Main outcomes of included studies that analyzed the association between active commuting to and from school (ACS) and academic achievement.

Reference	Results	Covariates
Ruiz-Hermosa et al. 2018 [40]	No differences were found between walking to school and passive commuters with language-related skills outcomes in children aged 4 to <6 years old. Walking to school was not associated with language-related skills and mathematics-related skills outcomes in children aged ≥6 to 7 years old.	Age, BMI, CRF, and SES.
García-Hermoso et al. 2017 [25]	Students who spent 30 to 60 min of ACS were more likely to have better scores in mathematics-related skills (OR = 2.19, 95% CI: 1.06 to 5.05; *p* = 0.028) and language-related skills (OR = 3.53, 95% CI: 1.12 to 4.37; *p* = 0.003) than noncommuters. No differences were found between non-ACS and ≤30 min of ACS groups or between non-ACS and >60 min of ACS groups.	Sex, weight status, birth weight, PA, screen time, maternal education, and SES.
Mora-González et al. 2017 [41]	Children in the non-ACS group had better scores than children in the ACS group in mathematics-related skills (7.46 ± 0.17 vs 6.95 ± 0.12, respectively; *p* = 0.009), language-related skills (7.72 ± 0.16 vs. 7.10 ± 0.12; *p* = 0.007), English (7.63 ± 0.17 vs. 7.01 ± 0.12; *p* = 0.002), natural sciences (7.59 ± 0.17 vs. 7.02 ± 0.12; *p* = 0.003) and overall academic achievement (7.60 ± 0.15 vs. 7.02 ± 0.11; *p* = 0.001). No differences were found between the ACS and non-ACS groups with academic achievement outcomes (mathematics-related skills, language-related skills, English, natural sciences, social sciences and overall academic achievement) in adolescents.	Age, sex, and school.
Ruiz-Ariza et al. 2017 [42]	Girls in the ACS group had significantly higher scores than girls in the non-ACS group in mathematics-related skills (6.47 ± 2.02 vs. 6.02 ± 2.15, respectively; *p* = 0.027), physical education (7.65 ± 1.38 vs. 7.28 ± 1.50; *p* = 0.005) and overall academic achievement (6.97 ± 1.49 vs. 6.58 ± 1.54; *p* = 0.008). No significant differences were found in boys.	Age and BMI.
Domazet et al. 2016 [43]	Students who cycled to and from school were more likely to have better scores than noncommuters in mathematics-related skills (OR = 5.4, 95% CI: 1.9 to 8.8; *p* < 0.01). Walking to and from school was not associated with mathematics-related skills.	Age, sex, SES, breakfast consumption, and supporting teaching outside the classroom during school hours.
Martins et al. 2016 [45]	No differences were found between non-ACS and one-way ACS or both-ways ACS in language-related skills, mathematics-related skills, and physical education outcomes.	Age, sex, SES, and school.
Van Dijk et al. 2014 [46]	No differences were found between ACS and passive commuters in mathematics-related skills and overall academic achievement outcomes.	Sex, academic year, SES, BMI, depressive symptoms, ethnicity, school level, and PA per week by accelerometer.
Stea and Torstveit 2014 [47]	ACS was positively associated with better scores in overall academic achievement than noncommuters in both girls (OR = 1.51, 95% CI: 1.10 to 2.08; *p* < 0.05) and boys (OR = 1.72, 95% CI: 1.26 to 2.35; *p* < 0.05).	BMI and SES.
Haapala et al. 2014 [48]	ACS was positively associated with reading fluency (β = 0.26, *p* < 0.01) and reading comprehension (β = 0.25, *p* < 0.01), but not with mathematics-related skills in boys. In girls, no associations were found.	Age, sex, SES, PANIC study group (exercise and diet vs. control), body fat percentage, lean body mass, CRF, motor performance, and reading disability.
Stock et al. 2012 [49]	ACS group was positively associated with higher overall academic achievement (OR = 2.03, 95% CI: 1.57 to 2.62).	SES and type of land use (buildings, single houses, farming, or traffic).
Martínez-Gómez et al. 2011 [50]	Girls in the active commuting to school group had significantly higher scores than girls in the non-active commuting to school group in language-related skills (20.82 ± 6.08 vs. 18.90 ± 5.88, respectively; *p* < 0.001) and mathematics-related skills (13.28 ± 4.91 vs. 12.36 ± 4.28; *p* = 0.008). In addition, girls in the active commuting to school > 15-minute group had better scores in mathematics-related skills than girls in the active commuting to school ≤ 15-minute group. No significant differences were found in boys.	Age, school, BMI, and extracurricular PA.

Abbreviations: BMI, body mass index; CRF, cardiorespiratory fitness; PA, physical activity; SES, socioeconomic status; CI, confidence interval; OR, odds ratio.

When studies provided linear regression beta coefficients or mean differences and standard deviations, these data were used to estimate the ES [51,52]. Additionally, when studies provided an odds ratio (OR), the ES was calculated with the natural log OR [51,53]. On the other hand, some statistical aspects that should be considered in this meta-analysis were as follows: (1) we separately estimated ES when studies provided data for children (aged 4 to <12 years) and adolescents (aged ≥12 to 18 years), boys and girls, and walking and cycling; (2) when studies included two or more follow-up measurements, only the baseline measurement was considered; (3) when studies presented several statistical adjustment models, we considered those that included the largest number of additional covariates; and (4) a pooled ES was only calculated when four or more studies included data of the same group of measured outcomes.

For each pooled ES, we conducted a sensitivity analysis by removing the studies one by one to assess the robustness of the summary estimates and to detect whether any particular study accounted for a large proportion of heterogeneity. In addition, subgroup analyses were based on the mode of ACS, age (children [aged 4 to <12 years] and adolescents [aged ≥12 to 18 years]) and sex.

Statistical analyses were performed using StataSE software, version 14 (StataCorp, College Station, TX, USA).

## 3. Results

### 3.1. Systematic Review

**Figure 1:**
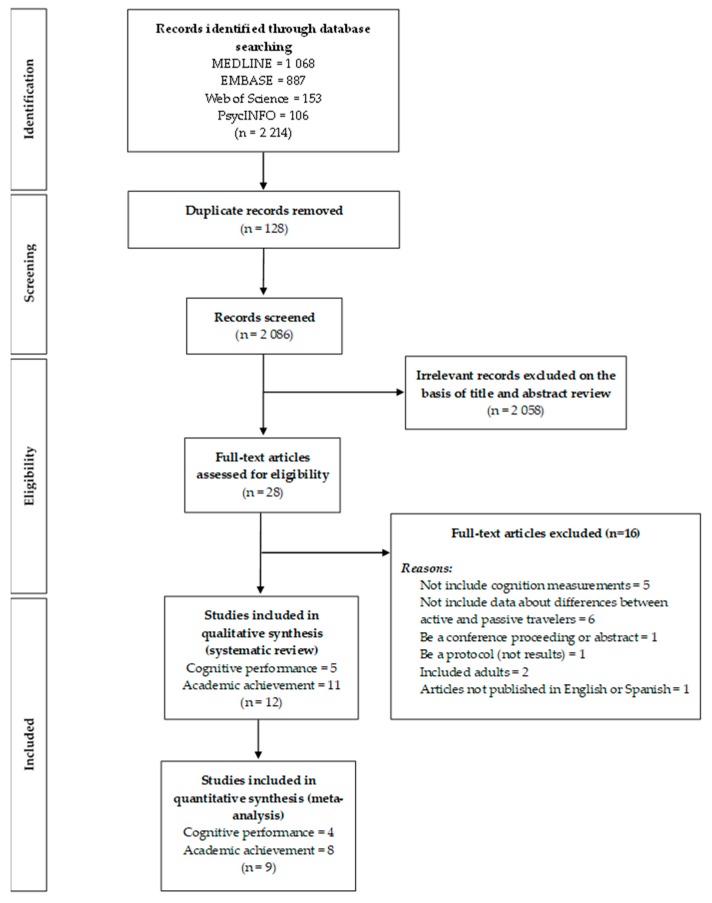
Flow diagram for the selection of studies of the association of commuting to and from school on cognition and academic achievement in children and adolescents, according to the Preferred Reporting Items for Systematic Review and Meta-Analysis (PRISMA).

The search retrieved a total of 2214 potentially eligible articles. After removing 128 duplicates, 2086 were screened in detail based on the title and abstract. Finally, a total of 12 studies met the inclusion criteria [25,40,41,42,43,44,45,46,47,48,49,50] (Figure 1).

The main characteristics of the included studies are summarized in Table 1. The studies were published between 2011 and 2018; they were conducted in seven European countries [40,41,42,43,44,45,46,47,48,49,50] and one in Chile [25]. Ten studies were cross-sectional studies [25,40,41,42,43,45,46,47,49,50] and two were follow-up studies [44,48]. The sample sizes of the studies ranged from 186 to 10,380 participants, and the total sample included 23,516 children and adolescents aged 4 to 18 years.

All the studies used self-report questions or diaries to assess ACS, with the exception of Van Dijk et al. [46] that used accelerometers. The studies showed a wide range of indicators or definitions to determine patterns of commuting to and from school and used different categories for differentiating between active and passive travelers (Table 1). Overall, researchers included information regarding the mode of commuting to and from school [25,40,41,42,43,44,45,47,48,49,50], duration of ACS [25,40,42,44,45,46,48,49,50], number of active trips on a weekly basis [42] and distance between home to school [45]. Finally, the mode of ACS varied across studies: three studies [25,40,42] included only information regarding walkers; eight studies [41,44,45,46,47,48,49,50] combined walkers with cyclists and skaters in the analyses; and only one study [43] analyzed cyclists and walkers separately.

Four articles analyzed both cognitive performance and academic achievement [40,43,46,50]. A total of five studies provided data regarding the cognitive performance of students for the following cognitive domains: nonexecutive cognitive functions [40], core executive functions [43,44,46] and metacognition [40,50]. Eleven articles provided data regarding the academic achievement of students. In these studies, researchers included information on marks for the following subjects: Mathematics-related skills (mathematics, arithmetic and numeracy) [25,40,41,42,43,45,46,48,50], language-related skills (language, vocabulary and writing) [25,40,41,42,45,46,50], reading [48], English (as a foreign language) [41,46], physical education [42,45], natural sciences [41], social sciences [41] and overall academic achievement (as grade point average or general perception of academic achievement) [41,42,47,49]. 

The evidence about the association between ACS and cognitive performance and academic achievement of included articles is summarized in Table 2 and Table 3, respectively. Among the studies that examined cognitive performance, two out of five studies found that ACS was associated with improvements in core executive functions [46] and metacognition [50] in adolescent girls but not in boys. Regarding academic achievement, a total of seven out of 11 studies found that ACS was associated with better marks in mathematics-related skills [25,42,43,50], language-related skills [25,50], reading [48], physical education [42], and overall academic achievement [42,47,49]. Of these studies, one article [43] found improvements only in cyclists, another study [48] showed positive significant differences only in boys and two studies [42,50] found better marks only in girls. Conversely, only one study [41] pointed out that ACS was inversely associated with academic achievement (mathematics-related skills, language-related skills, English, natural sciences and overall academic achievement).

Most studies reported models controlled for several covariates, showing a wide heterogeneity (Table 2 and Table 3). Seven studies included aged [40,41,42,43,45,48,50] and sex [25,41,43,44,45,46,48] of participants as a covariate in their analyses. While adiposity was included as a covariate in more than half of the studies [25,40,42,46,47,48,50], fitness was only included in two studies [40,48] and levels of PA of participants in three studies [25,46,50]. Nine studies [25,40,43,44,45,46,47,48,49] included the familial socioeconomic status (SES) as a possible variable of confusion. Finally, several studies included in this review also considered other potential confounders in their analysis: school [41,45,50], academic year [46], maternal education [25,44], birth weight [25], screen time [25], breakfast consumption [43], supporting teaching outside the classroom during school hours [43], residential neighborhood [44], air pollution [44], depressive symptoms [46], ethnicity [46], reading disability [48] and type of land use (buildings, single houses, farming, or traffic) [49]. 

### 3.2. Risk of Bias

Of the 12 studies in which risk of bias was assessed, 50% of the studies showed low risk, 16.7% showed medium risk, and 33.3% showed high risk (see supplementary material Table S3). All the studies showed a higher quality in the description of the study sample. However, only 33.3% of the studies showed an adequate adjustment of the main confounders. Similarly, 41.6% of the studies showed an adequate assessment/reporting of ACS. 

### 3.3. Meta-Analysis

A total of nine studies [25,40,41,42,43,44,45,47,50], which included data about differences between active and passive commuters on cognitive performance or academic achievement, were included in the meta-analysis.

#### 3.3.1. Cognitive Performance

**Figure 2:**
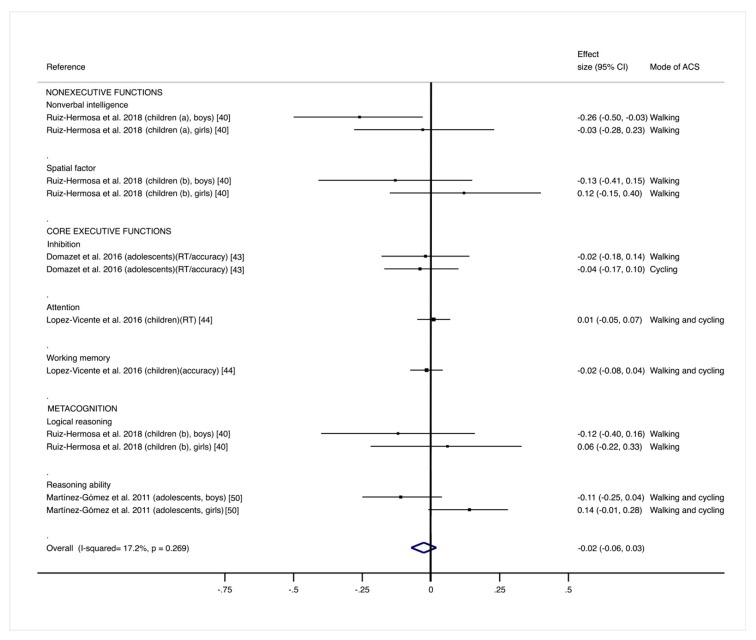
Pooled estimated effect size values for nonexecutive cognitive functions, core executive functions, and metacognition. Positive effect size values indicate higher score in outcomes in favor of the active commuting to and from school (ACS) group. Abbreviations: CI, confidence interval; RT, reaction time. (a) = children aged 4 to <6 years old; (b) = children aged ≥6 to 7 years old.

The pooled ES estimate was not statistically significant (ES: −0.02; 95% CI: −0.0.6 to 0.03) for the association between the mode of commuting to and from school (ACS or non-ACS) and a global measure of all cognitive domains (including nonexecutive cognitive functions, core executive functions, and metacognition) [40,43,44,50], and there was no important heterogeneity among studies (I^2^ = 17.2%; *p* = 0.269) (Figure 2).

#### 3.3.2. Academic Achievement

**Figure 3:**
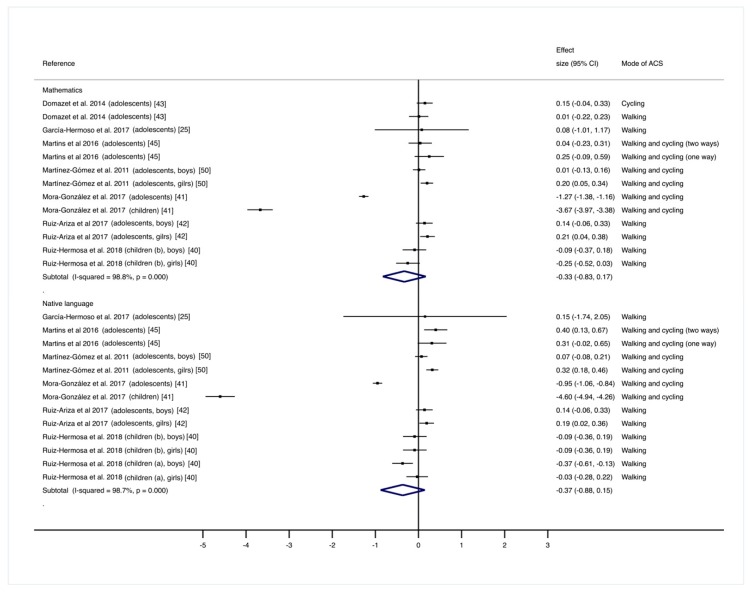
Pooled estimated effect size values for mathematics-related skills and language-related skills. Positive effect size values indicate higher score in outcomes in favor of the active commuting to and from school (ACS) group. Abbreviations: CI, confidence interval. (a) = children aged 4 to <6 years old; (b) = children aged ≥6 to 7 years old.

The pooled ES estimates for the relationship between the mode of commuting to and from school (ACS or non-ACS) on academic achievement was not statistically significant for mathematics-related skills [25,40,41,42,43,45,50] (ES: −0.33; 95% CI: −0.83 to 0.17) and language-related skills [25,40,41,42,45,50] (ES: −0.37; 95% CI: −0.88 to 0.15). Heterogeneity among the studies was considerable for mathematics-related skills (I^2^ = 98.8%; *p* = 0.001) and language-related skills (I^2^ = 98.7%; *p* = 0.001) (Figure 3).

#### 3.3.3. Sensitivity and Subgroup Analysis

Both the pooled ES estimates and heterogeneity were not significantly modified for mathematics-related skills, language-related skills and cognitive performance when the data from individual studies were removed from the analysis one by one (see supplementary material Table S4). In addition, subgroup analyses were based on the mode of ACS, age (children [aged 4 to <12 years] and adolescents [aged ≥12 to 18 years]) and sex did not show significant differences in the pooled ES estimates for mathematics-related skills and language-related skills (see supplementary material Table S5).

## 4. Discussion

To our knowledge, this is the first systematic review and meta-analysis that summarizes the evidence regarding the relationship between ACS and children´s and adolescents’ cognitive performance and academic achievement. Our findings suggest that ACS was not significantly associated with cognitive performance and academic achievement. However, there was insufficient evidence to draw a definitive conclusion due to the small number of studies and the level of heterogeneity among them.

Previous systematic reviews and meta-analyses have shown that PA has a positive impact on cognitive performance and academic achievement in children and adolescents [3,5,6,7,28,54]. Overall, this relationship seems to be determined by several mechanisms, such as increased oxygen and blood flow to the brain resulting in enhanced synaptic plasticity and neurogenesis [11,12,13]. Similarly, previous studies have shown that regular PA could produce changes in structural brain volumes and improve brain functioning [7,55,56]. However, despite the amount of evidence of these benefits, the results of this review are not enough to support a relationship between ACS and cognitive performance and academic achievement. Several factors and methodological weaknesses in the included studies could underlie these inconsistencies.

First, the studies included in this review showed a wide range of definitions to determine patterns of commuting to and from school and differentiate between active and passive commuters. On the one hand, some studies asked about the usual mode of commuting to and from school, whereas others only asked about the usual mode of commuting to school (but not from school) or by the number of active weekly trips. On the other hand, the studies used different categories for the mode of commuting to and from school, duration of commuting, and the number of active trips that counted as ACS. Along these lines, a recent systematic review provided a standardized self-report measure for this area of research to facilitate replication and comparison between future studies [57]. Additionally, future studies should also consider the use of more objective instruments to measure commuting to and from school (e.g., accelerometry, wearable daily movement tracking or positioning systems) to shed more light on these relationships.

On the other hand, most studies included in this review showed low frequencies of participants who cycled to and from school or combined walking and cycling in their analysis. Indeed, only Domazet et al. [43] examined cycling separately, and they reported that while cycling to and from school was positively associated with a greater mathematics performance, walking to and from school had no positive impact on performance in this subject. Previous studies have suggested that light, moderate and vigorous PA are associated with differential improvements in academic performance [3,58,59]. However, other studies have described a situation in which only PA with a particular intensity seems to have a crucial role with improvements in cognitive functioning [3,7]. Therefore, it is possible that different levels of PA intensity are associated with diverse effects on academic achievement and cognitive performance [3]. In this sense, it is conceivable to speculate that the intensity of walking was not sufficient to produce improvements in academic achievement and cognitive performance in children and adolescent at these ages. Therefore, it is not surprising that other reviews that have analyzed the relationship of ACS with variables such as fitness or adiposity have not found consistent results for schoolchildren who walked to and from school but did find a positive impact for cyclists [26,27].

In addition, a high proportion of the studies included in this review were conducted in Spain, where rates of cycling to and from school are uncommon, and students usually prefer walking to and from school [60]. Moreover, although commuting distance to and from school is the most common factor that can determine the decision to actively commute [26,61,62], none of the studies in this review included the distance of the commute to and from school as a covariate in their analyses. Along these lines, two studies [25,50] in this review showed that a longer duration (an indirect indicator of distance) of ACS may have had a positive influence on academic achievement and cognitive performance in children and adolescents. Thus, it is essential for future studies to expand our understanding of the optimal distance and duration of ACS that are necessary to produce improvements in cognitive performance and academic achievement.

Similarly, only half of the included studies in this review [40,42,46,47,48,50] analyzed the association of ACS with academic achievement or cognitive performance by sex subgroups. Stea and Torstveit [47] showed that higher values of academic achievement were associated with ACS in boys and girls among the adolescent population. However, three studies [42,46,50] found that ACS was positively associated with better cognitive performance and academic achievement in adolescent girls but not in adolescent boys. Among populations of children, Ruiz-Hermosa et al. [40] found that ACS was not associated with cognitive performance in either boys or girls, whereas Haapala et al. [48] reported a positive association in the academic performance of boys but not girls. Therefore, our results suggest that it is possible that there is a sex difference in the relationship between ACS with cognitive performance and academic achievement in the case of adolescents. Some of the reasons that could explain this sex-specific effect are differences in PA levels between boys and girls produced during adolescence, the neurotrophic hypothesis of depression, the stress of school or other changes specifically produced during adolescence [46,50].

Another aspect that should be considered is that several studies included in this review did not consider different potential confounders in their analysis, such as age, extracurricular PA, or the surrounding environment. Along these lines, SES has been identified as an important factor that can determine the decision to actively commute [26,61]. In addition, previous research has indicated that the SES of families has a strong association with academic achievement of young people and could mediate the relationship between PA and academic performance [58,63]. Indeed, a growing body of literature has revealed associations between SES and some neurocognitive domains as well as the structure and function of the brain [64]. This fact could explain the results of some research, as in the case of the study conducted by Mora-Gonzalez et al. [41], who found that ACS was inversely associated with academic achievement in Spanish children. The adiposity and fitness of the participants could be another confounding variable in the relationship between ACS and cognition and academic achievement. In this review, seven studies [25,40,42,46,47,48,50] included adiposity as a covariate, while fitness was only included in two studies [40,48]. Several studies have indicated that overweight/obesity are related to structural alterations in the brain and negatively associated with cognitive performance and academic achievement in children and adolescents [65,66]. Moreover, higher levels of physical fitness have also been linked to a positive effect on cognitive function and academic achievement [12,28,55,66]. Consequently, these and other possible confounders, such as air pollution or genetic factors, should be studied and taken into account in future studies for this area of research. 

### Limitations

Regarding this review, there are several limitations that should be considered when interpreting these results. First, we should consider that this review only included studies with a cross-sectional design or the baseline measurements of cohort studies. However, to our knowledge, there are no intervention studies that analyze the effects of ACS on cognitive performance or academic achievement. Second, it is necessary to point out that the meta-analyses were conducted using ES estimates and their corresponding 95% CIs from the results of each study, but not using the original data as provided by the studies (i.e., OR values or beta coefficients). In addition, it is possible that studies with poor or nonsignificant results were less likely to be published. Thus, bias cannot be ruled out. Third, few studies included analysis of subgroups by sex and age, which could have influenced our findings. However, the sensitivity analyses by subgroups did not show significant ES differences. On the other hand, the methodological differences between the studies (for example, the wide range of definitions to determine patterns of commuting to and from school or the different scales and tools for measuring cognitive performance and academic achievement), the lack of studies controlling for potential confounders (such as total PA, distance from home to school or SES) in their analyses, and the small number of studies included in this review could influence the generalizability of our results. Thus, the results should be interpreted with caution. Lastly, it is possible publication bias since our search strategy was limited to published studies in English or Spanish and did not include conference abstracts or grey literature.

## 5. Conclusions

This is the first systematic review and meta-analysis that provides summarized evidence regarding the relationship of ACS on children´s and adolescents’ cognitive performance and academic achievement. Our results show that there was no consistent evidence regarding the relationship between ACS and cognitive performance and academic achievement. Future studies are needed using a standardized self-reports or objective measure of ACS to determine consistently the effect of ACS on cognitive performance and academic achievement in children and adolescents. These studies should accurately assess the characteristics of the ACS (energy expenditure demands, duration and distance), because these characteristics may confuse the influence of ACS on academic achievement and cognitive performance. The control of mediators and confounders such as age, sex, total PA, SES, or weight status and fitness must be another key aspect to consider in future research. In addition, it is essential to separately analyze walking, cycling or even other means of commuting (such as skates or scooters) to and from school since their characteristics in terms of intensity and type of exercise could have different impacts on cognitive performance and academic achievement.

## Supplementary Materials

The following are available online at https://www.mdpi.com/1660-4601/16/10/1839/s1: Table S1: MOOSE checklist for meta-analyses of observational studies; Table S2: Search strategy for MEDLINE; Table S3: Methodological quality of the included studies; Table S4: Sensitivity analysis involving the removal of studies one by one for mathematics-related skills, language-related skills, and cognitive performance.; Table S5: Subgroup analyses based on the mode of commuting to and from school, age, and sex for mathematics-related skills and language-related skills.

## References

[1] Janssen I., Leblanc A.G. (2010). Systematic review of the health benefits of physical activity and fitness in school-aged children and youth. Int. J. Behav. Nutr. Phys. Act..

[2] Poitras V.J., Gray C.E., Borghese M.M., Carson V., Chaput J., Janssen I., Katzmarzyk P.T., Pate R.R., Gorber S.C., Kho M.E. (2016). Systematic review of the relationships between objectively measured physical activity and health indicators in school-aged children and youth. Appl. Physiol. Nutr. Metab..

[3] Esteban-Cornejo I., Tejero-Gonzalez C.M., Sallis J.F., Veiga O.L. (2015). Physical activity and cognition in adolescents: A systematic review. J. Sci. Med. Sport.

[4] Savina E., Garrity K., Kenny P., Doerr C. (2016). The Benefits of Movement for Youth: A Whole Child Approach. Contemp. Sch. Psychol..

[5] Álvarez-Bueno C., Pesce C., Cavero-Redondo I., Sánchez-López M., Martínez-Hortelano J.A., Martínez-Vizcaíno V. (2017). The Effect of Physical Activity Interventions on Children’s Cognition and Metacognition: A Systematic Review and Meta-Analysis. J. Am. Acad. Child Adolesc. Psychiatry.

[6] Álvarez-Bueno C., Pesce C., Cavero-Redondo I., Sánchez-López M., Garrido-Miguel M., Martínez-Vizcaíno V. (2017). Academic Achievement and Physical Activity: A Meta-analysis. Pediatrics.

[7] Tomporowski P.D., Davis C.L., Miller P.H., Naglieri J.A. (2009). Exercise and childrens intelligence, cognition, and academic achievement. Educ. Psychol. Rev..

[8] Ahn S., Fedewa A.L. (2011). A Meta-analysis of the Relationship Between Children’s Physical Activity and Mental Health. J. Pediatr. Psychol..

[9] Papacharisis V., Goudas M., Danish S.J., Theodorakis Y. (2005). The effectiveness of teaching a life skills program in a sport context. J. Appl. Sport Psychol..

[10] Pesce C., Marchetti R., Forte R., Crova C., Scatigna M., Goudas M., Danish S.J. (2016). Youth Life Skills Training: Exploring Outcomes and Mediating Mechanisms of a Group-Randomized Trial in Physical Education. Sport. Exerc. Perform. Psychol..

[11] Singh A., Uijtdewilligen L., Twisk J.W.R., van Mechelen W., Chinapaw M.J.M. (2012). Physical Activity and Performance at School: A Systematic Review of the Literature Including a Methodological Quality Assessment. Arch. Pediatr. Adolesc. Med..

[12] Hillman C.H., Erickson K.I., Kramer A.F. (2008). Be smart, exercise your heart: Exercise effects on brain and cognition. Nat. Rev. Neurosci..

[13] Diamond A.B. (2015). The Cognitive Benefits of Exercise in Youth. Curr. Sports Med. Rep..

[14] Singh A.S., Saliasi E., van den Berg V., Uijtdewilligen L., de Groot R.H.M., Jolles J., Andersen L.B., Bailey R., Chang Y.K., Diamond A. (2019). Effects of physical activity interventions on cognitive and academic performance in children and adolescents: A novel combination of a systematic review and recommendations from an expert panel. Br. J. Sports Med..

[15] Kovács E., Siani A., Konstabel K., Hadjigeorgiou C., De Bourdeaudhuij I., Eiben G., Lissner L., Gwozdz W., Reisch L., Pala V. (2014). Adherence to the obesity-related lifestyle intervention targets in the IDEFICS study. Int. J. Obes..

[16] Hallal P.C., Andersen L.B., Bull F.C., Guthold R., Haskell W., Ekelund U., Alkandari J.R., Bauman A.E., Blair S.N., Brownson R.C. (2012). Global physical activity levels: Surveillance progress, pitfalls, and prospects. Lancet.

[17] Konstabel K., Veidebaum T., Verbestel V., Moreno L.A., Bammann K., Tornaritis M., Eiben G., Molnár D., Siani A., Sprengeler O. (2014). Objectively measured physical activity in European children: the IDEFICS study. Int. J. Obes..

[18] Karnik S., Kanekar A. (2012). Childhood obesity: A global public health crisis. Int. J. Prev. Med..

[19] Roman-Viñas B., Zazo F., Martínez-Martínez J., Aznar-Laín S., Serra-Majem L. (2018). Results From Spain’s 2018 Report Card on Physical Activity for Children and Youth. J. Phys. Act. Health.

[20] Cabeza R., Kingstone A. (2008). Handbook of Functional Neuroimaging of Cognition.

[21] Spear L.P. (2013). Adolescent Neurodevelopment. J. Adolesc. Health.

[22] Herting M.M., Chu X. (2017). Exercise, cognition, and the adolescent brain. Birth Defects Res..

[23] Cairney J., Veldhuizen S., Kwan M., Hay J., Faught B.E. (2004). Biological age and sex-related declines in physical activity during adolescence. Med. Sci. Sport. Exerc..

[24] Chillón P., Ortega F.B., Ruiz J.R., Veidebaum T., Oja L., Mäestu J., Sjöström M. (2010). Active commuting to school in children and adolescents: An opportunity to increase physical activity and fitness. Scand. J. Soc. Med..

[25] García-Hermoso A., Saavedra J.M., Olloquequi J., Ramírez-Vélez R. (2017). Associations between the duration of active commuting to school and academic achievement in rural Chilean adolescents. Environ. Health Prev. Med..

[26] Larouche R., Saunders T.J., Faulkner G.E.J., Colley R., Tremblay M. (2014). Associations between active school transport and physical activity, body composition, and cardiovascular fitness: a systematic review of 68 studies. J. Phys. Act. Health.

[27] Lubans D.R., Boreham C.A., Kelly P., Foster C.E. (2011). The relationship between active travel to school and health-related fitness in children and adolescents: a systematic review. Int. J. Behav. Nutr. Phys. Act..

[28] Donnelly J.E., Hillman C.H., Castelli D.M., Etnier J.L., Lee S., Tomporowski P., Lambourne K., Szabo-reed A.N. (2016). Physical activity, fitness, cognitive function, and academic achievement in children: a systematic review. Med. Sci. Sport. Exerc..

[29] Tomporowski P.D., McCullick B., Pendleton D.M., Pesce C. (2015). Exercise and children’s cognition: The role of exercise characteristics and a place for metacognition. J. Sport Health Sci..

[30] Keeley T.J.H., Fox K.R. (2009). The impact of physical activity and fitness on academic achievement and cognitive performance in children. Int. Rev. Sport Exerc. Psychol..

[31] Diamond A. (2014). Executive Functions. Annu. Rev. Clin. Psychol..

[32] Stroup D.F., Berlin J.A., Morton S.C., Olkin I., Williamson G.D., Rennie D., Moher D., Becker B.J., Sipe T.A., Thacker S.B. (2000). Meta-analysis of observational studies in epidemiology: A proposal for reporting. J. Am. Med. Assoc..

[33] Cochrane Handbook for Systematic Reviews of Interventions Version 5.1.0. www.handbook.cochrane.org.

[34] Von Elm E., Altman D.G., Egger M., Pocock S.J., Gøtzsche P.C., Vandenbroucke J.P. (2007). The Strengthening the Reporting of Observational Studies in Epidemiology (STROBE) Statement: Guidelines for Reporting Observational Studies. PLoS Med..

[35] Armijo-Olivo S., Stiles C.R., Hagen N.A., Biondo P.D., Cummings G.G. (2012). Assessment of study quality for systematic reviews: a comparison of the Cochrane Collaboration Risk of Bias Tool and the Effective Public Health Practice Project Quality Assessment Tool: methodological research. J. Eval. Clin. Pract..

[36] Smith S., Madden A.M. (2016). Body composition and functional assessment of nutritional status in adults: a narrative review of imaging, impedance, strength and functional techniques. J. Hum. Nutr. Diet..

[37] Rodriguez-Ayllon M., Cadenas-Sánchez C., Estévez-López F., Muñoz N.E., Mora-Gonzalez J., Migueles J.H., Molina-García P., Henriksson H., Mena-Molina A., Martínez-Vizcaíno V. (2019). Role of Physical Activity and Sedentary Behavior in the Mental Health of Preschoolers, Children and Adolescents: A Systematic Review and Meta-Analysis. Sport. Med..

[38] Cohen J. (1988). Statistical Power Analysis for the Behavioral Sciences.

[39] DerSimonian R., Kacker R. (2007). Random-effects model for meta-analysis of clinical trials: An update. Contemp. Clin. Trials.

[40] Ruiz-Hermosa A., Martínez-Vizcaíno V., Alvarez-Bueno C., García-Prieto J.C., Pardo-Guijarro M.J., Sánchez-López M. (2018). No Association Between Active Commuting to School, Adiposity, Fitness, and Cognition in Spanish Children: The MOVI-KIDS Study. J. Sch. Health.

[41] Mora-Gonzalez J., Rodríguez-López C., Cadenas-Sanchez C., Herrador-Colmenero M., Esteban-Cornejo I., Huertas-Delgado F.J., Ardoy D.N., Ortega F.B., Chillón P., Mora-Gonzalez J. (2017). Active commuting to school was inversely associated with academic achievement in primary but not secondary school students. Acta Paediatr..

[42] Ruiz-Ariza A., De La Torre-Cruz M.J., Suárez-Manzano S., Martínez-López E.J. (2017). Active commuting to school influences on academic performance of Spanish adolescent girls. Retos.

[43] Domazet S.L., Tarp J., Huang T., Gejl A.K., Andersen L.B., Froberg K., Bugge A. (2016). Associations of physical activity, sports participation and active commuting on mathematic performance and inhibitory control in adolescents. PLoS ONE.

[44] Lopez-Vicente M., Forns J., Esnaola M., Suades-Gonzalez E., Alvarez-Pedrerol M., Robinson O., Julvez J., Garcia-Aymerich J., Sunyer J., López-Vicente M. (2016). Physical activity and cognitive trajectories in schoolchildren. Pediatr. Exerc. Sci..

[45] Martins J., Sallis J.F., Marques A., Diniz J., da Costa F.C. (2016). Potential correlates and outcomes of active commuting to school among adolescents. Motricidade.

[46] Van Dijk M.L., De Groot R.H.M., Van Acker F., Savelberg H.H.C.M., Kirschner P.A. (2014). Active commuting to school, cognitive performance, and academic achievement: an observational study in Dutch adolescents using accelerometers. BMC Public Health.

[47] Stea T.H., Torstveit M.K. (2014). Association of lifestyle habits and academic achievement in Norwegian adolescents: a cross-sectional study. BMC Public Health.

[48] Haapala E.A., Poikkeus A.M., Kukkonen-Harjula K., Tompuri T., Lintu N., Vaisto J., Leppanen P.H.T., Laaksonen D.E., Lindi V., Lakka T.A. (2014). Associations of physical activity and sedentary behavior with academic skills-a follow-up study among primary school children. PLoS ONE.

[49] Stock C., Bloomfield K., Ejstrud B., Vinther-Larsen M., Meijer M., Gronbaek M., Grittner U. (2012). Are characteristics of the school district associated with active transportation to school in Danish adolescents?. Eur. J. Public Health.

[50] Martinez-Gomez D., Ruiz J.R., Gomez-Martinez S., Chillon P., Rey-Lopez J.P., Diaz L.E., Castillo R., Veiga O.L., Marcos A., Martínez-Gómez D. (2011). Active commuting to school and cognitive performance in adolescents: The AVENA study. Arch. Pediatr. Adolesc. Med..

[51] Lipsey M.W., Wilson D.B. (2001). Practical Meta-Analysis.

[52] Peterson R.A., Brown S.P. (2005). On the use of beta coefficients in meta-analysis. J Appl Psychol..

[53] Chinn S. (2000). A simple method for converting an odds ratio to effect size for use in meta-analysis. Stat. Med..

[54] Fedewa A.L., Ahn S. (2011). The Effects of Physical Activity and Physical Fitness on Children’s Achievement and Cognitive Outcomes: a meta-analysis. Res. Q. Exerc. Sport.

[55] Chaddock-heyman L., Hillman C.H., Cohen N.J., Kramer A.F. (2014). III. The importance of physical activity and aerobic fitness for cognitive control and memory in children. Monogr. Soc. Res. Child Dev..

[56] Hillman C.H., Pontifex M.B., Castelli D.M., Khan N.A., Raine L.B., Scudder M.R., Drollette E.S., Moore R.D., Wu C.-T., Kamijo K. (2014). Effects of the FITKids Randomized Controlled Trial on Executive Control and Brain Function. Pediatrics.

[57] Herrador-Colmenero M., Pérez-García M., Ruiz J.R., Chillón P. (2014). Assessing modes and frequency of commuting to school in youngsters: A systematic review. Pediatr. Exerc. Sci..

[58] Kwak L., Kremers S.P.J., Bergman P., Ruiz J.R., Rizzo N.S., Sjo M. (2009). Associations between Physical Activity, Fitness, and Academic Achievement. J. Pediatr..

[59] Morales J., Gomis M., Pellicer-Chenoll M., García-Massó X., Gómez A., González L.-M. (2011). Relation between Physical Activity and Academic Performance in 3rd-Year Secondary Education Students. Percept. Mot. Skills.

[60] Brug J., Van Stralen M.M., Velde S.J., Chinapaw M.J.M., De I., Lien N., Bere E., Maskini V., Singh A.S., Maes L. (2012). Differences in Weight Status and Energy-Balance Related Behaviors among Schoolchildren across Europe: The ENERGY-project. PLoS ONE.

[61] Davison K.K., Werder J.L., Lawson C.T. (2008). Children ’ s Active Commuting to School: Current Knowledge and Future Directions. Rev. Lit. Arts Am..

[62] Ramírez-Vélez R., Beltrán C.A., Correa-Bautista J.E., Vivas A., Prieto-Benavidez D.H., Martínez-Torres J., Triana-Reina H.R., Villa-González E., Garcia-Hermoso A. (2016). Factors associated with active commuting to school by bicycle from Bogotá, Colombia: The FUPRECOL study. Ital. J. Pediatr..

[63] Coe D.P., Peterson T., Blair C., Schutten M.C., Peddie H. (2013). Physical Fitness, Academic Achievement, and Socioeconomic Status in School-Aged Youth. J. Sch. Health.

[64] Farah M.J. (2017). The neuroscience of socioeconomic status: Correlates, causes, and consequences. Neuron Rev..

[65] Reinert K.R.S., Po E.K., Barkin S.L. (2013). The Relationship between Executive Function and Obesity in Children and Adolescents: A Systematic Literature Review. J. Obes..

[66] Torrijos-Niño C., Martínez-Vizcaíno V., Pardo-Guijarro M.J., García-Prieto J.C., Arias-Palencia N.M., Sánchez-López M. (2014). Physical fitness, obesity, and academic achievement in schoolchildren. J. Pediatr..

