# Supplementary material for: Active Commuting to and from School, Cognitive Performance, and Academic Achievement in Children and Adolescents: A Systematic Review and Meta-Analysis of Observational Studies
Source: Int J Environ Res Public Health. 2019 May 23;16(10):1839. doi: 10.3390/ijerph16101839 (PMC6571885; doi:10.3390/ijerph16101839)
Supplement: Supplementary file 1 [file ijerph-16-01839-s001.pdf]

International Journal of
Environmental Research and Public Health

MDPI

Table S1. MOOSE checklist for meta-analyses of observational studies.

Item No	Recommendation	Reported on Page No
	Reporting of background should include	
1	Problem definition	1-2
2	Hypothesis statement	1-2
3	Description of study outcome(s)	1-2
4	Type of exposure or intervention used	1-2
5	Type of study designs used	3
6	Study population	3
	Reporting of search strate.g.y should include	
7	Qualifications of searchers (e.g., librarians and investigators)	4
8	Search strategy, including time period included in the synthesis and key words	3
9	Effort to include all available studies, including contact with authors	4
10	Databases and registries searched	13
11	Search software used, name and version, including special features used (e.g., explosion)	12
12	Use of hand searching (e.g., reference lists of obtained articles)	3
13	List of citations located and those excluded, including justification	13
14	Method of addressing articles published in languages other than English	3
15	Method of handling abstracts and unpublished studies	3
16	Description of any contact with authors	12
	Reporting of methods should include	
17	Description of relevance or appropriateness of studies assembled for assessing the hypothesis to be tested	3
18	Rationale for the selection and coding of data (e.g., sound clinical principles or convenience)	3
19	Documentation of how data were classified and coded (e.g., multiple raters, blinding and interrater reliability)	3
20	Assessment of confounding (e.g., comparability of cases and controls in studies where appropriate)	3-4
21	Assessment of study quality, including blinding of quality assessors, stratification or regression on possible predictors of study results	3-4
22	Assessment of heterogeneity	4
23	Description of statistical methods (e.g., complete description of fixed or random effects models, justification of whether the chosen models account for predictors of study results, dose-response models, or cumulative meta-analysis) in sufficient detail to be replicated	4,12
24	Provision of appropriate tables and graphics	5-11
	Reporting of results should include	
25	Graphic summarizing individual study estimates and overall estimate	15-16
26	Table giving descriptive information for each study included	4-10
27	Results of sensitivity testing (e.g., subgroup analysis)	14
28	Indication of statistical uncertainty of findings	14
	Reporting of discussion should include	
29	Quantitative assessment of bias (e.g., publication bias)	14
30	Justification for exclusion (e.g., exclusion of non-English language citations)	18
31	Assessment of quality of included studies	15-18
	Reporting of conclusions should include	
32	Consideration of alternative explanations for observed results	17
33	Generalization of the conclusions (i.e., appropriate for the data presented and within the domain of the literature review)	18
34	Guidelines for future research	16
35	Disclosure of funding source	19

Table S2. Search strategy for Medline.

"commuting”<br>OR<br>“active commuting"<br>OR<br>"active commuting to school"<br>OR<br>"active commuting from school"<br>OR<br>“active transportation to school”<br>OR<br>“active transportation from school”<br>OR<br>“walk*”<br>OR<br>“walking to school”<br>OR<br>“walking from school”<br>OR<br>“cycling”<br>OR<br>“cycling to school”<br>OR<br>“cycling from school”<br>OR<br>“bicycling”<br>OR<br>“bicycling to school”<br>OR<br>“bicycling from school”<br>OR<br>“skateboarding”<br>OR<br>“skateboarding to school”<br>OR<br>“skateboarding from school”<br>OR<br>“lifestyle habit*”	AND	“cognition”<br>OR<br>“executive”<br>OR<br>“executive function”<br>OR<br>“academic”<br>OR<br>“academic skill*”<br>OR<br>“academic achievement”<br>OR<br>“academic performance”<br>OR<br>“academic behavior*”<br>OR<br>“academic grade *”<br>OR<br>“cognitive performance”<br>OR<br>“cognitive control”<br>OR<br>“cognitive flexibility”<br>OR<br>“intelligence”<br>OR<br>“memory”<br>OR<br>“attention”<br>OR<br>“mathematic performance”<br>OR<br>“inhibitory control”<br>OR<br>“working memory”<br>OR<br>“decision making”<br>OR<br>“metacognition”	AND	“children”<br>OR<br>“childhood”<br>OR<br>“pre-schooler”<br>OR<br>“schoolchildren”<br>OR<br>“preadolescent”<br>OR<br>“adolescent*”<br>OR<br>“adolescence”

The symbol * indicates the truncation of a word.

Table S3. Methodological quality of the included studies.

Reference [a]	Item 1	Item 2	Item 3	Item 4	Item 5	Total Score [b]
Ruiz-Hermosa et al. 2018 [40]	1	1	1	0	1	4
García-Hermoso et al. 2017 [25]	1	1	1	1	1	5
Mora-González et al. 2017 [41]	1	0	1	0	0	2
Ruiz-Ariza et al. 2017 [42]	1	1	1	0	1	4
Domazet et al. 2016 [43]	1	0	1	0	1	3
López-Vicente et al. 2016 [44]	1	0	1	0	0	2
Martins et al. 2016 [45]	1	1	0	1	1	4
Van Dijk et al. 2014 [46]	1	1	1	1	1	5
Stea and Torstveit 2014 [47]	1	0	1	0	1	3
Haapala et al. 2014 [48]	1	0	1	1	1	4
Stock et al. 2012 [49]	1	0	0	0	0	1
Martínez-Gómez et al. 2011 [50]	1	0	1	0	0	2

Risk of bias score was calculated based on the five criteria based on the STrengthening the Reporting of OBservational studies in Epidemiology (STROBE) criteria [34] and the Effective Public Health Practice Project (EPHPP) [35]. Item 1 adequate description of the study sample (number of participants, mean age and sex). Item 2 adequate assessment/reporting of ACS (ACS measurement was clearly defined and validated, and the studies included at least three of the following data: duration/distance, intensity, frequency or analysis separating walking, cycling or other means of commuting to school). Item 3 adequate assessment of the cognitive performance and academic achievement outcomes (validity/reliability of the outcome measure reported and/or measurement procedure adequately described). Item 4 adequate adjustment of confounders (the studies considered at least three of the following confounding variables: sex, age, familial socioeconomic status, distance or total physical activity). Item 5 description of both the numbers and reasons for withdrawals and dropouts (participation rate at baseline at least 70%). [a] All the studies were cross-sectional, except for López-Vicente et al. [44] and Haapala et al. [48] that were follow-up studies. [b] The scores were summed to provide a total score out of 5, using the following cate.g.ories: 0-2 “high risk”, 3 “medium risk”, and 4-5 “low risk”.

Table S4. Sensitivity analysis involving the removal of studies one by one for mathematics-related skills, language-related skills and cognitive performance.

**Mathematics-related skills**			
**Reference**	**ES**	**95% CI**	**I-squared**
Domazet et al. 2014 (adolescents) [43]	-0.37	-0.91 to 0.17	98.8
Domazet et al. 2014 (adolescents) [43]	-0.36	-0.89 to 0.18	98.8
García-Hermoso et al. 2017 (adolescents) [25]	-0.35	-0.87 to 0.16	98.9
Martins et al 2016 (adolescents) [45]	-0.36	-0.89 to 0.17	98.8
Martins et al 2016 (adolescents) [45]	-0.38	-0.90 to 0.15	98.8
Martínez-Gómez et al. 2011 (adolescents, boys) [50]	-0.36	-0.91 to 0.20	98.8
Martínez-Gómez et al. 2011 (adolescents, girls) [50]	-0.37	-0.92 to 0.17	98.8
Mora-González et al. 2017 (adolescents) [41]	-0.25	-0.72 to 0.23	98.2
Mora-González et al. 2017 (children) [41]	-0.05	-0.42 to 0.32	97.6
Ruiz-Ariza et al 2017 (adolescents, boys) [42]	-0.37	-0.91 to 0.17	98.8
Ruiz-Ariza et al 2017 (adolescents, girls) [42]	-0.38	-0.91 to 0.16	98.8
Ruiz-Hermosa et al. 2018 (children aged 6-7 years old; boys) [40]	-0.35	-0.88 to 0.18	98.9
Ruiz-Hermosa et al. 2018 (children aged 6-7 years old; girls) [40]	-0.34	-0.87 to 0.20	98.9
**Language-related skills**			
**Reference**	**ES**	**95% CI**	**I-squeared**
García-Hermoso et al. 2017 (adolescents) [25]	-0.39	-0.91 to 0.14	98.8
Martins et al 2016 (adolescents) [45]	-0.43	-0.97 to 0.11	98.8
Martins et al 2016 (adolescents) [45]	-0.42	-0.96 to 0.12	98.8
Martínez-Gómez et al. 2011 (adolescents, boys) [50]	-0.40	-0.98 to 0.17	98.8
Martínez-Gómez et al. 2011 (adolescents, girls) [50]	-0.43	-0.99 to 0.14	98.7
Mora-González et al. 2017 (adolescents) [41]	-0.31	-0.86 to 0.23	98.5
Mora-González et al. 2017 (children) [41]	-0.01	-0.32 to 0.31	96.4
Ruiz-Ariza et al 2017 (adolescents, boys) [42]	-0.41	-0.97 to 0.15	98.8
Ruiz-Ariza et al 2017 (adolescents, girls) [42]	-0.41	-0.98 to 0.15	98.8
Ruiz-Hermosa et al. 2018 (children aged 6-7 years old, boys) [40]	-0.39	-0.94 to 0.16	98.8
Ruiz-Hermosa et al. 2018 (children aged 6-7 years old, girls) [40]	-0.39	-0.94 to 0.16	98.8
Ruiz-Hermosa et al. 2018 (children 4-6 years old, boys) [40]	-0.36	-0.92 to 0.19	98.8
Ruiz-Hermosa et al. 2018 (preschool 4-6 years old, girls) [40]	-0.39	-0.95 to 0.16	98.8
**Cognitive performance**			
**Reference**	**ES**	**95% CI**	**I-squeared**
Ruiz-Hermosa et al. 2018 (children 4-6 years old, boys) [40]	-0.01	-0.04 to 0.03	0.0
Ruiz-Hermosa et al. 2018 (children 4-6 years old, girls) [40]	-0.02	-0.06 to 0.03	25.2
Ruiz-Hermosa et al. 2018 (children aged 6-7 years old, boys) [40]	-0.01	-0.06 to 0.03	21.1
Ruiz-Hermosa et al. 2018 (children aged 6-7 years old, girls) [40]	-0.02	-0.06 to 0.03	20.0
Domazet et al. 2016 (adolescents, walking)(RT/accuracy) [43]	-0.02	-0.07 to 0.03	25.2
Domazet et al. 2016 (adolescents, cycling)(RT/accuracy) [43]	-0.01	-0.06 to 0.04	24.2
Lopez-Vicente et al. 2016 (children)(RT) [44]	-0.03	-0.08 to 0.03	21.1
Lopez-Vicente et al. 2016 (children)(accuracy) [44]	-0.02	-0.08 to 0.04	25.1
Ruiz-Hermosa et al. 2018 (children aged 6-7 years old, boys) [40]	-0.01	-0.06 to 0.03	21.8
Ruiz-Hermosa et al. 2018 (children aged 6-7 years old, girls) [40]	-0.02	-0.07 to 0.03	23.8
Martínez-Gómez et al. 2011 (adolescents, boys) [50]	-0.01	-0.05 to 0.04	12.9
Martínez-Gómez et al. 2011 (adolescents, girls) [50]	-0.02	-0.06 to 0.02	0.0

Abbreviations: ES, effect size; CI, confidence interval; RT, reaction time.

Table S5. Subgroup analyses based on the mode of commuting to and from school, age and sex for mathematics-related skills and language-related skills.

**Mathematics-related skills**				
**Subgroup analysis**	**n**	**ES**	**95% CI**	**I-squared**
**Mode of commuting**				
Cycling	1	0.15	-0.04 to 0.33	-
Walking	6	0.03	-0.11 to 0.18	49.2
Cycling and walking	6	-0.74	-0.16 to 0.18	99.4
**Age**				
Children	3	-1.34	-3.57 to 0.90	99.5
Adolescents	10	-0.03	-0.45 to 0.40	98.0
**Sex**				
Boys	3	0.03	-0.07 to 0.14	1.1
Girls	3	0.08	-0.15 to 0.31	77.9

**Language-related skills**				
**Subgroup analysis**	**N**	**ES**	**95% CI**	**I-squared**
**Mode of commuting**				
Cycling	0	-	-	-
Walking	8	-0.62	-1.52 to 0.29	99.0
Cycling and walking	6	-0.74	-1.71 to 0.23	99.4
**Age**				
Children	5	-1.03	-2.50 to 0.43	99.3
Adolescents	8	0.07	-0.38 to 0.51	97.7
**Sex**				
Boys	4	-0.05	-0.26 to 0.16	75.8
Girls	4	0.13	-0.06 to 0.31	70.8

Abbreviations: ES, effect size; CI, confidence interval.
